# Editorial: Clinical and surgical perspectives in sublobar resection for lung cancer

**DOI:** 10.3389/fsurg.2026.1833090

**Published:** 2026-04-01

**Authors:** Takuya Fujita

**Affiliations:** Division of General Thoracic Surgery, Kohka Public Hospital, Kohka, Japan

**Keywords:** lung cancer, lymph node metastasis, QOL (quality of life), segmentectomy, sublober resection

We are pleased to release this Research Topic of “*Clinical and Surgical Perspectives in Sublobar Resection for Lung Cancer*”. We would like to express our deepest gratitude to all the authors who contributed their valuable research and to the reviewers for their insightful comments. Our special thanks go to the editorial board for their dedication to maintaining high academic standards.

In this Research Topic, we feature a series of articles on the latest advancements in Sublobar Resection for Lung Cancer. We hope that these insights will provide valuable, actionable knowledge for your clinical practice.

## Five highly excellent papers were published

Magouliotis et al. conducted a systematic review of five studies involving 1,149 cases to compare lobectomy with sublobar resection and stereotactic body radiotherapy. They reported that sublobar resection resulted in significantly better postoperative quality of life compared to lobectomy.

Zhu et al. demonstrated that postoperative radiotherapy (PORT) may improve overall survival (OS) in patients with resectable pN2 stage IIIA non-small cell lung cancer, particularly those with more than one lymph node metastasis. These findings suggest that PORT may yield favorable treatment outcomes in patients with extensive lymph node metastasis, indicating a need for further prospective studies to validate and expand these observations.

Huang et al. demonstrated that methods such as positive lymph node count (nPLN), lymph node ratio (LNR), log odds ratio for positive lymph nodes (LODDS) are used to predict prognosis in non-small cell lung cancer (NSCLC) patients. Compared to nPLN and LNR staging systems, LODDS demonstrated superior prognostic predictive ability in stage I to IIIA NSCLC patients undergoing lobectomy or pneumonectomy.

Ye et al. developed a non-invasive, interpretable biomarker combining clinical and radiomic features from preoperative CT to accurately identify airspace diffusion (STAS) in clinically stage IA lung adenocarcinoma using artificial intelligence and CT signatures. The CT-based radiomic model demonstrated good diagnostic performance in predicting STAS in clinically stage IA lung adenocarcinoma.

Huang et al. conducted a comparative evaluation of negative lymph node count, positive lymph node count, and lymph node ratio in predicting survival outcomes after complete resection of non-small cell lung cancer (NSCLC). They identified negative lymph node count (NLN), positive lymph node count (NPLN), and lymph node ratio (LNR) as independent prognostic indicators in NSCLC patients. Among these, the predictive model based on NLN showed slightly superior prognostic predictive value compared to NPLN, while NPLN demonstrated better performance than the LNR model. Notably, high NLN and low NPLN/LNR values were consistently associated with improved survival prognosis. The association between these prognostic markers and NSCLC survival rates requires further validation through prospective studies.

This topic has yielded insights into sublober resection from various perspectives. We believe it will significantly contribute to future clinical practice.

We hope you find this issue informative and inspiring.

Takuya Fujita

Topic Editor, Clinical and Surgical Perspectives in Sublobar Resection for Lung Cancer

